# Supplementary material for: Extracellular Vesicles Derived from Human Umbilical Mesenchymal Stem Cells Transfected with miR-7704 Improved Damaged Cartilage and Reduced Matrix Metallopeptidase 13
Source: Cells. 2025 Jan 9;14(2):82. doi: 10.3390/cells14020082 (PMC11763736; doi:10.3390/cells14020082)
Supplement: Supplementary file 1 [file cells-14-00082-s001.zip › cells-3417688-supplementary.pptx]

## Slide 1
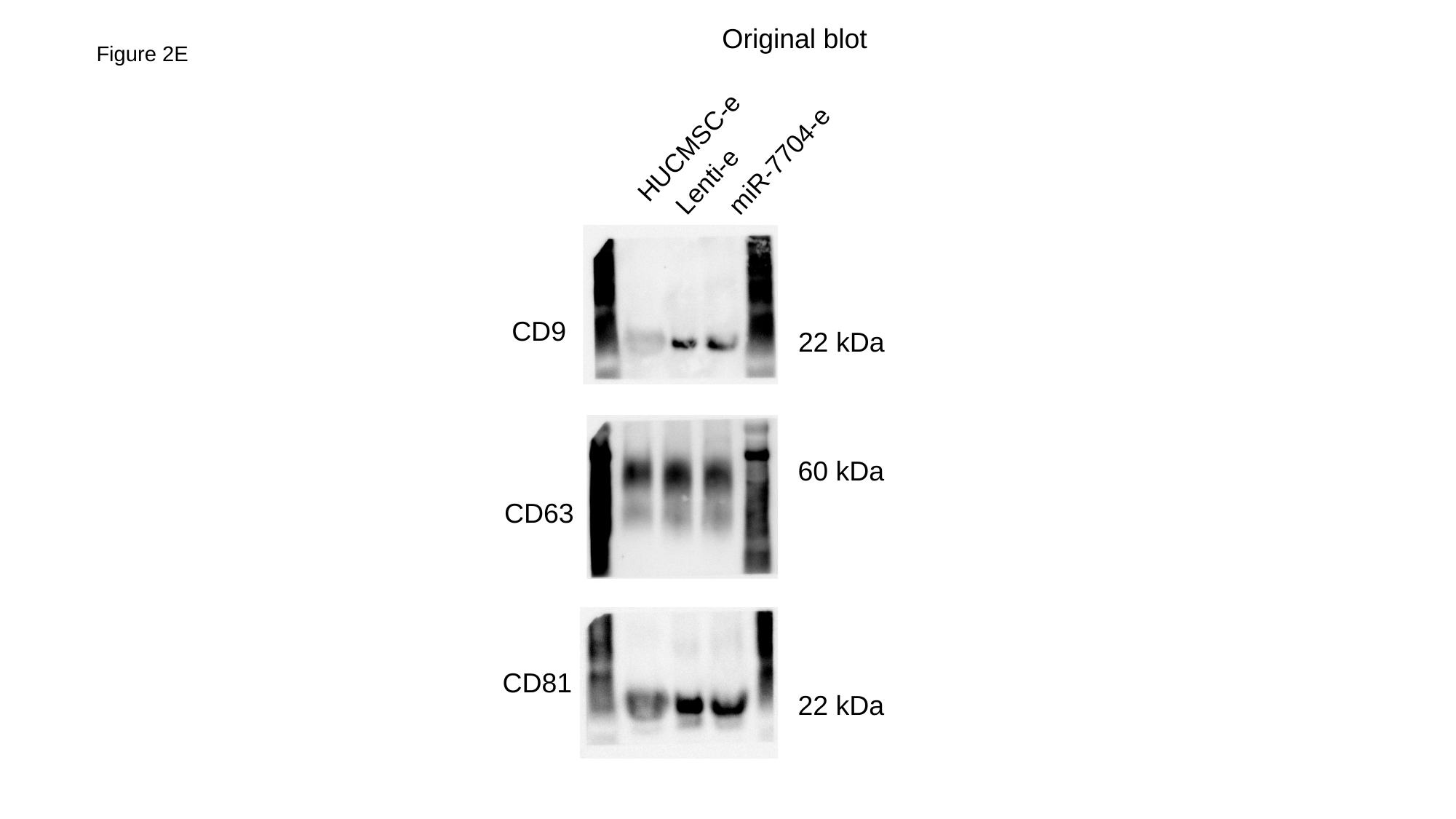

Original blot
Figure 2E
HUCMSC-e
miR-7704-e
Lenti-e
CD9
22 kDa
60 kDa
CD63
CD81
22 kDa

## Slide 2
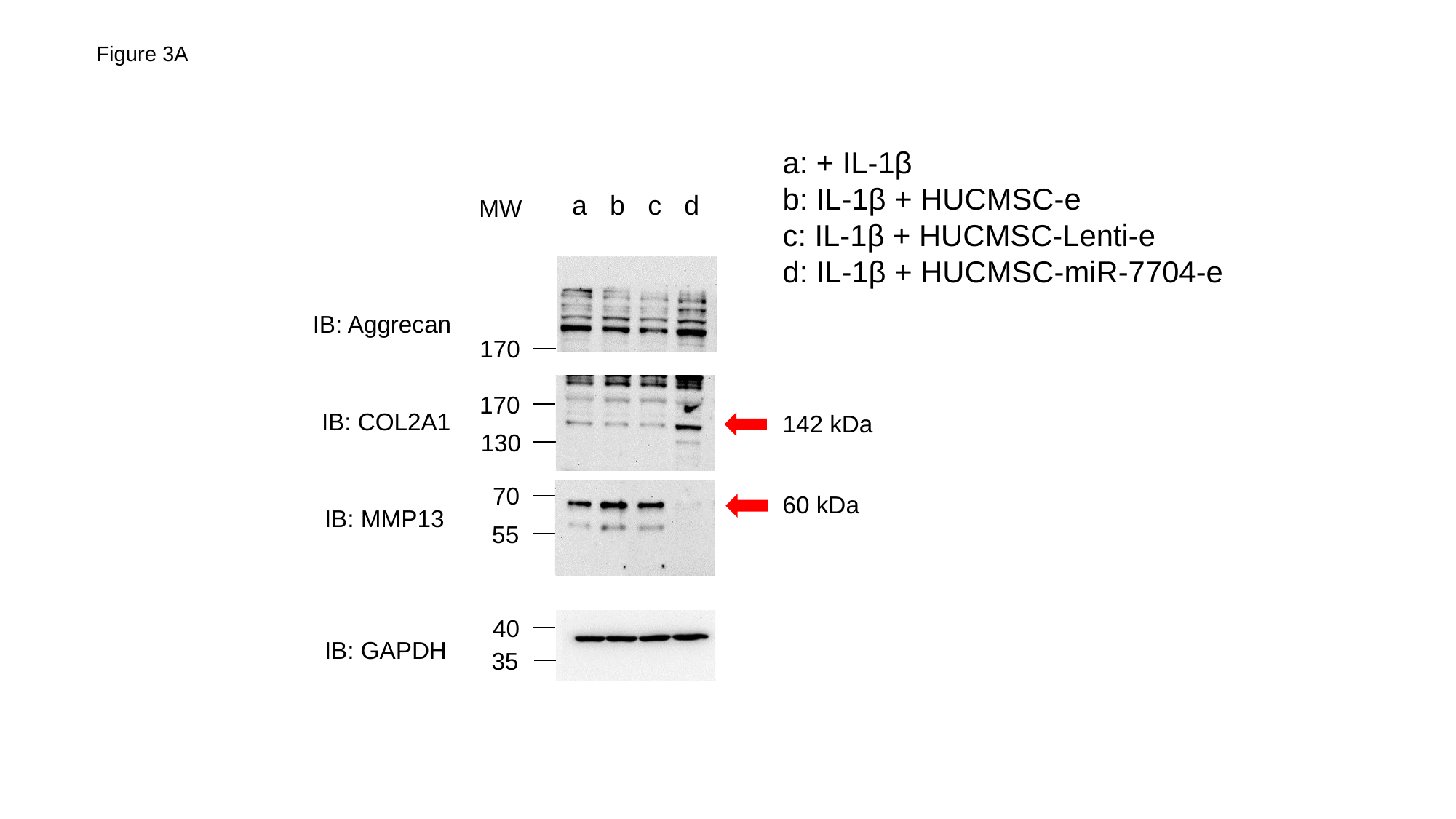

Figure 3A
a: + IL-1β
b: IL-1β + HUCMSC-e
c: IL-1β + HUCMSC-Lenti-e
d: IL-1β + HUCMSC-miR-7704-e
a b c d
MW
IB: Aggrecan
170
170
IB: COL2A1
142 kDa
130
70
60 kDa
IB: MMP13
55
40
IB: GAPDH
35
